# Cadmium, Environmental Exposure, and Health Outcomes

**DOI:** 10.1289/ehp.0901234

**Published:** 2009-10-05

**Authors:** Soisungwan Satarug, Scott H. Garrett, Mary Ann Sens, Donald A. Sens

**Affiliations:** Department of Pathology, University of North Dakota School of Medicine and Health Sciences, Grand Forks, North Dakota, USA

**Keywords:** cadmium, calcium, cancer, diet, disease burden, environmental exposure, iron, manganese, zinc

## Abstract

**Objectives:**

We provide an update of the issues surrounding health risk assessment of exposure to cadmium in food.

**Data sources:**

We reviewed epidemiologic studies published between 2004 and 2009 concerning the bioavailability of cadmium in food, assessment of exposure, and body burden estimate, along with exposure-related effects in nonoccupationally exposed populations.

**Data extraction and synthesis:**

Bioavailability of ingested cadmium has been confirmed in studies of persons with elevated dietary exposure, and the findings have been strengthened by the substantial amounts of cadmium accumulated in kidneys, eyes, and other tissues and organs of environmentally exposed individuals. We hypothesized that such accumulation results from the efficient absorption and systemic transport of cadmium, employing multiple transporters that are used for the body’s acquisition of calcium, iron, zinc, and manganese. Adverse effects of cadmium on kidney and bone have been observed in environmentally exposed populations at frequencies higher than those predicted from models of exposure. Increasing evidence implicates cadmium in the risk of diseases that involve other tissues and organ systems at cadmium concentrations that do not produce effects on bone or renal function.

**Conclusions:**

Population data raise concerns about the validity of the current safe intake level that uses the kidney as the sole target in assessing the health risk from ingested cadmium. The data also question the validity of incorporating the default 5% absorption rate in the threshold-type risk assessment model, known as the provisional tolerable weekly intake (PTWI), to derive a safe intake level for cadmium.

Because of its high rates of soil-to-plant transfer, cadmium is a contaminant found in most human foodstuffs, which renders diet a primary source of exposure among nonsmoking, nonoccupationally exposed populations (Clemens 2006; Franz et al. 2008; McLaughlin et al. 2006). A safe intake limit of 7 μg cadmium/week/kg body weight was set based on the critical renal cadmium concentration of between 100 and 200 μg/g wet weight that corresponds to a urinary threshold limit of 5–10 μg/g creatinine [World Health Organization (WHO) 1989, 1993]. However, numerous studies have revealed adverse kidney effects at urinary cadmium levels < 0.5 μg/g creatinine (Satarug and Moore 2004). Further, accumulating evidence links environmental exposure to cadmium with increased cancer incidence. For example, in prospective studies in Japan and the United States, excess cancer mortality was found to be associated with environmental exposure to cadmium (Arisawa et al. 2007b; Menke et al. 2009; Nishijo et al. 2006). Åkesson et al. (2008) observed increased endometrial cancer risk in a Swedish cohort among participants who consumed > 15 μg/day of cadmium, mainly from cereals and vegetables. These findings suggest a very large health burden associated with exposure to cadmium at levels experienced by many populations worldwide.

This review provides an update on cadmium exposure levels and the potential adverse health effects they may elicit in adult populations. We focus first on key issues underpinning health risk assessment of low-level cadmium in the diet, including bioavailability of dietary origin, the 5% default absorption rate, thresholds and safe intake levels, and the kidney as a specific target for cadmium accumulation. Second, we review epidemiologic studies from 2004 to 2009 that link exposure levels to observed effects in classic targets (kidney and bone) along with newly identified potential target organs. We also summarize evidence that links cadmium with diabetes, diabetic nephropathy, hypertension, peripheral artery disease (PAD), myocardial infarction, diminished lung function, periodontal disease, and age-related macular degeneration (AMD). Evidence from prospective studies reveal potential causal relationships of cadmium exposure with life prognosis (all-cause mortality) and excess cancer mortality and suggest that cadmium is at least a comorbidity factor if not a causative factor. Specifically, we summarize cadmium-cancer associations for the lung, pancreas, breast, endometrium, prostate, and urinary bladder.

## FAO/WHO Guidelines for Safe Intake

The major issue addressed in this article is whether the guidelines established for the safe intake of cadmium adequately protect individuals from increased health risk. The Food and Agriculture Organization/World Health Organization (FAO/WHO) Joint Expert Committee on Food Additives has defined the provisional tolerable weekly intake (PTWI) for a chemical with no intended function as an estimate of the amount of the chemical that can be ingested weekly over a lifetime without appreciable health risk (WHO 1989). The PTWI value initially set for cadmium was 400–500 μg/person/week (WHO 1989). These levels were based on a critical renal concentration of 100–200 μg cadmium/g wet kidney cortex weight, attained after a cadmium intake of 140–260 μg/day for > 50 years or 2,000 mg over a lifetime (WHO 1989). The PTWI model incorporates an oral absorption rate of 5% and a daily excretion rate of 0.005% of total body burden. In 1992, the PTWI for cadmium was refined and subsequently expressed in terms of cadmium intake per kilogram of body weight (WHO 1993). This refinement also recognized that the model PTWI for cadmium did not include a safety factor and that only a very modest margin existed between the level of exposure in a normal diet and a level predicted to produce a potential effect on the kidney. Despite this narrow safety factor, the PTWI for cadmium was retained at 7 μg/kg body weight, which translates to 70 μg/day for a person who weighs 70 kg. A toxicokinetic model predicts, based on similar assumptions, that the renal cortical cadmium level of 50 μg/g wet weight could be attained at the cadmium intake of 1 μg/kg body weight/day over 50 years (Buchet et al. 1990). The renal cortical cadmium 50 μg/g wet weight corresponds to urinary cadmium 2 μg/g creatinine, but kidney effects have been observed at urinary cadmium levels < 0.5 μg/g creatinine (Table 1). These findings argue that the current safe intake level does not provide sufficient health protection and that it should be lowered.

Satarug et al. (2000, 2003) examined the PTWI model by studying cadmium accumulation in kidneys and livers of environmentally exposed subjects. Their studies suggested that the safe intake level for an adult should be < 30 μg/day. They also showed that cadmium accumulation in the kidney cortex increased with age, reaching a plateau by 50 years of age (Satarug et al. 2002). An estimated dietary intake at 25–30 μg cadmium/day for persons in the 41- to 50-year-old age group would give rise to a total cadmium body burden of 18 mg. The studies indicated that the estimated intake of 25–30 μg/day may produce adverse kidney effects in about 1% of the adult population when variability in absorption and sensitivity to adverse effects among population members are considered in the analysis.

## Threshold-based Models for Safe Intake

If the relative susceptibility of humans and animals is unknown at the time of derivation of PTWI, the lowest observed adverse effect level (LOAEL) in the most sensitive species is used, which adds an uncertainty factor of 100. Thus, the PTWI value must be substantiated by additional experimental data, and, if warranted, a larger uncertainty factor should be applied to the value. An alternative to LOAEL, the benchmark dose (BMD), has been used to derive the urinary cadmium threshold. The BMD is defined as the exposure level that produces a change in a response, known as the point of departure (POD). The lower 95% confidence interval (CI) of the BMD corresponding to a 5% (L5) or 10% (L10) level of each index of an adverse effect above the background level may also be calculated as a threshold. Uno et al. (2005) estimated the BMDL10 of urinary cadmium to be 0.6–1.2 μg/g creatinine (0.8–1.6 μg/day) in men and 1.2–3.6 μg/g creatinine (0.5–4.7 μg/day) in women. These results were based on data from 828 Japanese subjects (410 men, 418 women), 40–59 years of age, who lived in areas without apparent pollution. In another study, Suwazono et al. (2006) used data from 790 Swedish women, 53–64 years of age, and estimated the BMD of urinary cadmium to be 0.6–1.1 μg/g creatinine. Data from selected studies found the POD for early kidney and bone effects to be between 0.5 and 3 μg/g creatinine (Järup and Åkesson 2009). Using the BMD-derived urinary cadmium threshold, the tolerable weekly intake for cadmium was 2.5 μg/kg body weight, which corresponds to 25 μg/day for a person who weighs 70 kg (European Food Safety Authority 2009).

## International Food Legislation

In the early 1960s, the Joint FAO/WHO Codex Alimentarius Commission was established to detail international food legislation. In 2000, the Codex Committee for Food Additives and Contaminants reached an agreement on the principles for setting maximum levels (MLs) for cumulative food contaminants (Francesconi 2007). MLs were proposed for lead (Pb^2+^) and cadmium (Cd^2+^) in various food categories, including rice, soybean, peanuts, and bivalve mollusks. The bioavailability and ML of cadmium became an issue because certain bivalve mollusks were known to be naturally high in cadmium content (Francesconi 2007). A high ML for cadmium was based on an early study on bluff oysters (McKenzie et al. 1986).

## Cadmium Sources and Bioavailability

### Mollusks and crustaceans

Bivalve mollusks and crustaceans are filter feeders that accumulate metals from the aquatic environment independent of environmental pollution, and contaminated waters could further increase the content of metals (Whyte et al. 2009). Cadmium content of some Pacific oysters was found to be 13.5 mg/kg dry weight, whereas 2-fold higher cadmium content was reported for some New Zealand bluff oysters (Copes et al. 2008). A bioavailability study was conducted on 57 men and 19 women 20–75 years of age who were associated with the oyster industry (McKenzie et al. 1986). The subjects were divided into groups 1, 2, 3, and 4, according to their average weekly oyster consumption rate at < 6, 6–24, 24 to < 72, and ≥ 72 oysters, respectively. The estimated cadmium intake for subjects in groups 1, 2, 3, and 4 was 34, 75, 116, and 250 μg/day, respectively. The estimated consumption for all groups, except group 1, exceeded the FAO/WHO safe guideline. The blood cadmium was higher among smokers than among nonsmokers. For the nonsmokers in group 4 (the highest consumption rate), the increase in blood cadmium attributable to oyster consumption was 1.2 μg/L. Blood selenium was also elevated by oyster consumption, but no effect on serum zinc or copper levels was observed. Urinary cadmium, zinc, and β2-microglobulin (β2-MG) levels were not affected, and no relationship was found between cadmium intake and adverse renal effects, defined as glycosuria or proteinuria. In addition, no effect was observed on levels of cadmium, zinc, and copper in hair. McKenzie et al. (1986) concluded that interactions with selenium and other metals in oysters may result in diminished cadmium absorption. This study is extremely important because it has been used as the basis for assigning high cadmium ML values to allow the marketing of oysters and their products that contain naturally high levels of cadmium. It is important to note that no distinction is made between toxicity of natural versus anthropogenic cadmium. In our opinion, this study had several flaws. For example, although dietary selenium and zinc were measured in the analysis, other determinants of cadmium absorption were not considered, such as body iron stores and the older age of the subjects. Furthermore, evidence (Tables 1–5) now indicates that the blood cadmium 1.2 μg/L attributed to oyster consumption among nonsmokers in group 4 can be considered at risk, because blood cadmium levels of < 1 μg/L have been associated with adverse effects.

Recently, Copes et al. (2008) and Clark et al. (2007) reexamined the bioavailability of cadmium in oysters and showed the effects of consuming oysters on cadmium body burden and serum elemental composition (selenium, zinc, copper). Copes et al. (2008) considered the potential confounding effects of age and cigarette smoking and restricted their study to nonsmokers (33 men, 28 women) between 33 and 64 years of age (mean age, 47.3 years). They estimated that the cadmium intake from oysters was 174 μg/week (24.8 μg/day). Significant increases in blood and urinary cadmium levels were found to be associated with the duration of oyster farming of at least 12 years during which time the on average consumption rate was 18 oysters/week (87 g/week). For the study participants, the average (range) blood cadmium was 0.83 (0.34–2.27) μg/L, and the average urinary cadmium (range) was 0.76 (0.16–4.04) μg/g creatinine. The mean urinary cadmium 0.76 μg/g creatinine was 2.5-fold greater than that of U.S. female nonsmokers, mean age 55 years, as defined in the study by McElroy et al. (2007). Cadmium in a shellfish diet was shown to be bioavailable in the study by Vahter et al. (1996) who found cadmium intake to be 11 μg/day for women in the mixed-diet group and 28 μg/day for those in the high-shellfish diet group. No differences in blood or urine cadmium levels were observed between the two groups. However, an increase in blood cadmium of 63% and an increase in urinary cadmium of 24% were found among those consuming the high-shellfish diet who had plasma ferritin levels < 20 μg/L when compared with those who consumed mixed diets and had the same low body iron stores. Thus, these studies strongly suggest that cadmium in oysters and shellfish is bioavailable and that long-term oyster consumption does result in a higher body burden of cadmium.

### Oilseeds

Sunflower seeds, peanuts, flaxseed, and linseed accumulate cadmium from the soil in a manner similar to that of tobacco. Cadmium levels in sunflower kernels range from 0.2 to 2.5 mg/kg. Reeves and Vanderpool (1997) conducted a study on 75 male and female nonsmokers who were 30–70 years of age. Using a self-reported food-frequency survey, those subjects who reported consuming > 28 g of sunflower kernels per week were considered high consumers. An analysis of a duplicate diet among controls showed that on average cadmium intake was 36 μg/day, but intake was not determined for any of the high consumers. Blood and urinary cadmium levels were used as indicators of cadmium body burden. The expected increased cadmium body burden could not be demonstrated, probably because of the limited number of subjects and the short time frame of the study. However, evidence for kidney effects, reflected by urinary β2-MG and *N*-acetyl-β-d-glucosaminidase (NAG) levels, was found among high consumers of sunflower seeds. These data may indicate that cadmium in sunflower kernels possess a high nephrotoxic potential. Alternatively, they may indicate increased sensitivity to cadmium renal toxicity in the high sunflower-kernel consumers.

### Offal

High cadmium levels (7–76 mg/kg wet weight) were found in the offal of dugongs and turtles that constituted the diet in the Torres Strait (Australia). Haswell-Elkins et al. (2007a) examined cadmium body burden in relation to offal consumption among residents in two communities with varying dugong and turtle catch statistics. Of the 182 subjects, 12% had urinary cadmium > 2 μg/g creatinine, and the group mean urinary cadmium was 0.83 μg/g creatinine. Age accounted for 46% of total variation in urinary cadmium levels, and sex (female) and current smokers accounted for 7% and 4.7% of variation, respectively. In a second study, Haswell-Elkins et al. (2007b) found high cadmium body burden associated with higher consumption of turtle liver and kidney and with locally gathered clams, peanuts, and coconuts. The sum of these foods, heavy smoking, age, and waist circumference accounted for 40% of variation in cadmium body burden (*p* < 0.05). Thus, this study showed that local offal consumption is linked with high cadmium body burden.

Cadmium levels are higher in liver and kidney than in muscle and older animals (Prankel et al. 2005). Average cadmium in the liver and kidney of wild moose was 2.11 and 20.2 μg/g wet weight, respectively (Arnold et al. 2006). Notably, chronic, low-dose exposure situations produce 10- to 20-fold higher cadmium in kidney than liver. It is worth noting that no difference was observed in bioavailability of ionic cadmium versus protein bound cadmium. In the human gastrointestinal tract, the protein bound to cadmium is digested and ionic cadmium released; thus speciation of cadmium in food would not be a basis for assigning high cadmium MLs for marketing purposes (Francesconi 2007). There has been no indication of decreases in food cadmium content over the past decade or any drastic change in dietary habits. In a British study, Lyon et al. (1999) showed that human kidney cadmium levels were static over a period of 16 years (1978–1993) but were higher than those found in studies conducted in the early 20th century. The distribution of kidney cadmium concentrations was skewed, with about 3.9% of the 2,700 samples > 50 μg/g kidney cortex wet weight, although the population mean was only 19 μg/g wet weight (Lyon et al. 1999).

## Cadmium Exposure and Effects Observed

### Kidney and bone: chronic high-dose effects

Long-term exposure to high-dose cadmium causes Itai-itai disease. This disease affects mainly women and is characterized by severely impaired tubular and glomerular function and generalized osteomalacia and osteoporosis that result in multiple bone fractures (Inaba et al. 2005). An estimate of cadmium intake, based on historic rice cadmium content, in the Itai-itai disease endemic area during the 1960s was 600 μg/day, and the threshold lifetime intake was estimated to be between 1,580 and 2,000 mg of cadmium (Kobayashi et al. 2002, 2006). In two reports, investigators showed that the lifetime threshold for early onset of the Itai-itai disease was less than a 3-fold difference from the intake observed in areas with no apparent pollution (Inaba et al. 2005; Uno et al. 2005). This may reflect a small safety margin between population intake levels and the levels that produce overt effects.

### Kidney and bone: chronic low-dose effects

Long-term exposure to low-dose cadmium has been linked to tubular impairment with a loss of reabsorptive capacity for nutrients, vitamins, and minerals. These losses include zinc and copper bound to the metal binding protein metallothionein (MT), glucose, amino acids, phosphate, calcium, β2-MG, and retinol-binding protein (RBP) [International Programme on Chemical Safety (IPCS) 1992]. The abnormal urinary excretion of low-molecular-weight proteins, calcium, amino acid, phosphate and glucose observed in cadmium-exposed individuals share some similarities with Fanconi’s syndrome, a genetic disorder of renal tubular transport. Urinary markers for cadmium effects are cadmium itself, low-molecular-weight substances, and the enzymes of renal tubular origin, such as NAG (Teeyakasem et al. 2007). In general, the urinary cadmium level reflects the body burden over long-term exposure before the development of kidney damage, and blood cadmium is considered an indicator of recent exposure (IPCS 1992). However, for persons > 60 years of age, blood cadmium is considered a better estimate of body burden than is urinary cadmium.

### Kidney and bone: the Cadmibel project

The Cadmibel study was one of the earliest investigations to examine the effects of low-dose exposure among 2,327 Belgian subjects between 1985 and 1989 (Buchet et al. 1990). The results demonstrated that there was a 10% probability of having tubular impairment when urinary cadmium levels exceeded 2–4 μg/day. The result was derived from a logistic regression of urinary cadmium and various markers, including urinary calcium, amino acids, NAG, RBP, and β2-MG. These markers demonstrated different thresholds for urinary cadmium levels. More than 10% of values for each marker were abnormal when urinary cadmium (micrograms per day) exceeded 1.92 for calcium, 2.74 for NAG, 2.87 for RBP, 3.05 for β2-MG, and 4.29 for amino acids. The findings showed that urinary calcium excretion increased by 10 mg/day for every 2-fold increment in urinary cadmium excretion. An increased susceptibility to cadmium among subjects with diabetes was noted.

### Kidney and bone: current exposure levels

Compelling evidence has linked tubular impairment with urinary calcium loss, rapid bone demineralization, and osteoporosis (Table 1). For example, Åkesson et al. (2005) showed that tubular impairment among women 53–64 years of age was associated with blood and urinary cadmium levels of 0.38 μg/L and 0.67 μg/g creatinine, respectively. Glomerular impairment was associated with urinary cadmium of 0.8 μg/g creatinine. In another study, Åkesson et al. (2006) used the same population and showed the body burden associated with decreased bone mineral density They also showed that participants with diabetes had increased susceptibility to the renal effects of cadmium and that menopausal women were more susceptible to cadmium-induced bone effects than were nonmenopausal women. The risk for osteoporosis among women ≥ 50 years of age increased by 43% when urinary cadmium levels were compared between groups with urinary cadmium < 0.5 and > 1.0 μg/g creatinine (Gallagher et al. 2008). In a prospective study of Flemish women, Schutte et al. (2008b) found bone effects among those with a 2-fold increase in body cadmium burden, but no tubular effects were documented in the population.

In Thailand, Satarug et al. (2005) found that tubular impairment and renal injury were associated with increased risk of high blood pressure among subjects 16–60 years of age who had mean urinary cadmium of 0.39 μg/L and mean serum cadmium of 0.47 μg/L. They demonstrated that a 3-fold increase in urinary cadmium (0.39 to 1.12 μg/L) was associated with an 11%, 32% and 61% increase in the probability of having high blood pressure, renal injury, and tubular impairment, respectively. The probability of having high blood pressure was increased by 20% among those with evidence of renal injury. The odds of tubular impairment were found to be 10.6 times higher when comparisons were made between urinary cadmium levels of 1–5 versus > 5 μg/g creatinine (Teeyakasem et al. 2007). Thomas et al. (2009) reported a dose–response relationship between urinary cadmium and early renal injury, whereas Wu et al. (2008) found progressive tubular and glomerular impairment among those with urinary cadmium > 10 μg/g creatinine. In a study of 14,778 U.S. adults > 20 years of age with mean blood cadmium and lead of 0.41 μg/L and 1.58 μg/L, respectively, Navas-Acien et al. (2009) found that the risk for albuminuria was 2.34; it was 1.98 for lowered glomerular filtration rate among those in the highest quartiles of blood cadmium and lead than among those in the lowest. These findings suggest that environmental exposure to cadmium and lead may constitute the risk factors for chronic kidney disease in the United States.

### Diabetes

Schwartz et al. (2003) demonstrated a dose response between urinary cadmium level and an increased risk of prediabetes and diabetes. The risk estimates for abnormal fasting glucose and diabetes were 1.48 and 1.24 when comparisons were made for urinary cadmium levels of < 1 with those between 1.00 and 1.99 μg/g creatinine. These values increased to 2.05 and 1.45 when they compared urinary cadmium < 1 μg/g with ≥ 2 μg/g creatinine, respectively. As noted by Edwards and Prozialeck (2009), the incidence of diabetes is rising globally and has reached epidemic levels in some nations. Thus, the potential role played by low-dose cadmium in prediabetes and diabetes warrants further research. In a study involving Chinese subjects between 44 and 78 years of age (mean, 66 years) with type 2 diabetes, Chen et al. (2006) found tubular impairment among those who had had diabetes for 8.6 years. They also noted that the risk for tubular impairment was increased by 3.34 when they compared urinary cadmium of < 1 versus ≥ 1 μg/g creatinine and by 5.56 when they compared low versus high levels of circulating MT antibody. These data suggested increased susceptibility to cadmium tubular effects among diabetic subjects with high MT antibody in plasma. The authors considered that mean urinary cadmium 0.38 μg/g creatinine and mean blood cadmium 0.61 μg/L were below threshold for glomerular effects. Afridi et al. (2008) reported higher blood and urinary cadmium among Pakistani men 31–60 years of age who had had type 2 diabetes, on average, for 16 years.

### Diabetic nephropathy

A dose–response relationship has been observed between urinary cadmium and albuminuria among Torres Strait subjects with type 2 diabetes (Haswell-Elkins et al. 2008). For persons with diabetes, the geometric mean for urinary cadmium with albuminuria was 61% higher than for those without albuminuria. For those without albuminuria, the average urinary cadmium level was 0.74 μg/g creatinine. The higher urinary cadmium levels among diabetic subjects could be the result of extensive kidney damage that leads to the release of cadmium in the kidney into the urine. One way to interpret these data is to suggest that the threshold urinary cadmium for people with diabetes should be no greater than 0.74 μg/g creatinine to prevent or delay the onset of renal complications. Such an interpretation considers albuminuria to be a predictor of glomerular impairment, end-stage renal failure, and adverse cardiovascular outcomes. A similar threshold was suggested in another study that found glomerular impairment associated with the urinary cadmium 0.8 μg/g creatinine (Åkesson et al. 2005).

### Hypertension

Eum et al. (2008) observed a dose–response relationship between urinary cadmium and hypertension. Of the Korean subjects in their study, 26.2% were hypertensive. For this population, the mean blood cadmium was 1.67μg/L, and the risk estimate for hypertension was 1.51 when blood cadmium levels in the lowest tertile were compared with those in the highest. An association was also found between blood cadmium and blood pressure levels in a U.S. sample population, where the mean blood cadmium was 3.98-fold lower than the mean level found in the Korean study (Tellez-Plaza et al. 2008). The strength of the cadmium blood pressure association was greatest among nonsmokers, intermediate among former smokers, and small or absent among current smokers. These findings support “pressor” effects, which have been shown to be characteristic of chronic exposure to low-dose cadmium (Satarug et al. 2005).

### Blood vessels and the heart

A set of studies has found evidence linking an increased risk of PAD with low-dose cadmium exposure (Navas-Acien et al. 2004, 2005). The risk for PAD was 1.07, 1.30, and 2.82 when blood cadmium quartiles 2, 3, and 4 were compared with the lowest quartile (*p* for trend = 0.01). Evidence that cadmium might be a key contributor to the high PAD risk was the finding that the risk of PAD for current smokers was 4.13-fold higher than for those who never smoked; for never smokers, the risk of PAD diminished to 1.84 after controlling for cadmium. In this study, subjects with PAD had 36% higher urinary cadmium than did those without disease where average urinary cadmium of the sample group was 0.36 μg/L and where the 25th and 90th percentile urinary cadmium level was 0.19 and 1.16 μg/L, respectively. Furthermore, the PAD risk was found to be 3.05 when the 75th percentile urinary cadmium was compared with that of the 25th percentile (Navas-Acien et al. 2005). It has also been shown that increased cadmium body burden is associated with lower aortic pulse wave velocity, lower pulse pressure, and higher femoral distensibility among subjects from low and high cadmium exposure areas (Schutte et al. 2008b). Everett and Frithsen (2008) found the risk of myocardial infarction among female subjects to be 1.8 when urinary cadmium > 0.88 μg/g creatinine was compared with < 0.43 μg/g creatinine. The risk remained when the analysis was restricted to nonsmokers.

### Lung

Lampe et al. (2008) examined the potential effects of exposure to cadmium on lung function using a sample group of 96 men who underwent one to three lung function tests between 1994 and 2002. They found a reduction in forced expiratory volume in 1 sec (a reflection of lung function) associated with increased urinary cadmium among those who smoked. These data suggest that lung disease among smokers may be mediated in part by cadmium, because urinary cadmium is also a marker of cumulative smoking, an established risk factor in lung disease.

### Periodontal tissues

A 3-fold increase in urinary cadmium (0.18 versus 0.63 μg/g creatinine) has been reported to be associated with a 54% higher prevalence odds ratio (OR) for periodontal disease. For example, Arora et al. (2009) found that among a sample of adults, 15.4% had periodontal disease. The age-adjusted mean urinary cadmium for subjects with periodontal disease was 0.50 μg/g creatinine and 0.30 μg/g creatinine for unaffected individuals.

### Ocular tissues

Higher urinary cadmium was found to be associated with AMD among smokers (Erie et al. (2007). The median urinary cadmium level of current and former smokers with AMD was 1.18 μg/g creatinine. This level was 1.97-, 2.03-, and 2.07-fold higher than that of smokers without AMD, nonsmokers with AMD, and nonsmokers without disease, respectively. Increased retinal cadmium content has also been found in male subjects with AMD (Wills et al. 2008, 2009).

### Mammary gland

Gundacker et al. (2007) showed that breast milk samples of Austrian subjects contained, on average, a cadmium content of 0.086 μg/L and that breast milk cadmium content was lower among nonsmokers who took vitamins and mineral supplements (*p* < 0.05). In a study by Kippler et al. (2008), the median cadmium level in breast milk from Bangladeshi subjects was 1.6-fold higher than was the level from Austrian subjects. The investigators observed a correlation between cadmium and the elemental composition of milk, including manganese, iron, and calcium levels. Their findings suggest a potential influence of cadmium on mammary gland metal transport and secretion.

### Cadmium and cancer

Cadmium is classified as a cancer-causing agent in humans based on an elevated incidence of lung cancer and mortality data derived from the occupational groups with evidence of elevated exposure to cadmium. Occupational exposures have historically been through inhalation [International Agency for Research on Cancer (IARC) 1993]. A consequence of this initial association of inhaled cadmium with cancer in occupationally exposed workers is that a carcinogenic risk from cadmium of dietary origin has long been ignored by regulatory agencies. However, literature to support a role for dietary cadmium that shows exposure levels associated with increased mortality risk and cancer mortality does exist as summarized in Table 4. In the Kakehashi cohort, a 2.5-fold increase in cancer mortality was observed among women with permanent tubular impairment (Nishijo et al. 2006). This study also noted increased mortality from nephritis, nephrosis, heart failure, and brain infarction among both men and women with severely impaired tubular function. Baseline median urinary cadmium values for men and women in the Kakehashi cohort were 7.0 and 12.1 μg/g creatinine, respectively. This cohort was also used to establish a dose response showing the lowest urinary cadmium of 3 μg/g creatinine associated with excess female mortality risk (Nakagawa et al. 2006). Similarly, Arisawa et al. (2007a) observed an increased mortality rate among subjects with permanent tubular impairment in the Nagasaki cohort I. They also observed a 2.58-fold concurrent increased risk of cancer mortality among those with tubular impairment. The determinants of increased mortality were renal injury, tubular impairment, and renal insufficiency. These effects of cadmium were absent in the Nagasaki cohort II study, most likely because of the selective loss of advanced cases and the reduction in exposure after soil restoration that was undertaken between 1980 and 1983 (Arisawa et al. 2007b). Of note, the cadmium exposure levels in the Kakehashi and the Nagasaki cohorts were close to the levels experienced by people in a cadmium pollution area in Thailand (Teeyakasem et al. 2007).

In contrast to the above studies, the cadmium exposure in a Belgian cohort and in a U.S. cohort was below the level that would cause renal injury and yet increased mortality was observed in these studies. In the Belgian cohort, Nawrot et al. (2008) observed a 20% increase in mortality in the low-exposure area. This percentage was increased by 44% in the high-exposure area. Further, mortality risks were increased by 25% and 33% among those with a 2-fold increase in blood cadmium who resided in low- and high-exposure areas, respectively. Menke et al. (2009) observed in the U.S. cohort, an increase in cancer mortality by 4.29-fold among men with urinary cadmium levels < 0.21 versus > 0.48 μg/g creatinine. They also observed a 1.68-fold increase in all-cause mortality among men after adjusting for cadmium exposure from cigarette smoking. Mean urinary cadmium for men in the U.S. cohort was 0.28 μg/g creatinine, which was 1.43-fold lower than that for women.

### Cadmium as a multitissue carcinogen

A substantial number of recent reports have noted a link between cadmium and cancer in nonoccupationally exposed populations (Table 5). In the 15-year Belgian cohort, Nawrot et al. (2006) observed a 1.7-, 4.2- and 1.57-fold increase in lung cancer risk among those with a 2-fold increase in cadmium body burden, those living in a high exposure area, and those with a 2-fold increase in soil-cadmium content, respectively. Serum cadmium and a farming occupation have been associated with pancreatic cancer with the risk attributed to increased serum cadmium of 1.12 μg/L and of 3.25 μg/L for farming occupation (Kriegel et al. 2006). A dose response between breast cancer risk and cadmium exposure could be seen when individuals with urinary cadmium of ≤ 0.26 were compared with those with ≥ 0.58 μg/g creatinine, suggesting a 2.29-fold increase in risk (McElroy et al. 2006). In a prospective study, Åkesson et al. (2008) found a 2.9-fold increase in endometrial cancer risk among women with cadmium intake greater than an average value of 15 μg/day; 80% of cadmium intake was derived from cereals and vegetables. Several studies have examined prostate disease. A dose–response relationship was shown between urinary cadmium and abnormal serum levels of prostate specific antigen (PSA) (Zeng et al. 2004). It has also been shown that an increase in urinary cadmium to 1 μg/g creatinine is associated with a 35% increase in serum PSA level among men whose zinc intakes were < 12.7 mg/day (van Wijngaarden et al. 2008). Safe and adequate zinc intake for an adult is 15 mg/day (Slikker et al. 2004). A 4.7-fold increase in prostate cancer risk was found among subjects where toenail cadmium was compared between individuals with < 0.007 and among those with > 0.03 μg cadmium/g toenail (Vinceti et al. 2007). In a study of bladder cancer, Kellen et al. (2007) demonstrated a 5.7-fold increase in risk between subjects with blood cadmium at the lowest tertile versus the highest. The risk estimate was corrected for sex, age, smoking habits, and workplace exposure. Mean blood cadmium for bladder cancer cases was 1.1 μg/L and this level was 1.6-fold higher than that of the controls.

## Cadmium Body Burden

### Sex and tissue differential cadmium accumulation

Tissue collected from postmortem examinations has been used to define cadmium accumulation levels in tissues and organs of human subjects (Table 6). In an analysis of 61 environmentally exposed subjects between 2 and 89 years of age (mean 38.5 years), Satarug et al. (2002) revealed that renal cadmium accumulation was greater in younger age groups with little increase, or even a reduction, in the older age groups. Some investigators have suggested that younger individuals have high rates of renal cadmium accumulation because of a very high rate of dietary cadmium absorption (Horiguchi et al. 2004; Kikuchi et al. 2003). Conversely, a lack of renal cadmium accumulation in older individuals may be caused by a fall in dietary absorption rate plus a reduction in tubular reabsorptive capacity, which is associated with the aging of the kidney. A few studies have examined sex differences and cadmium accumulation. For example, Satarug et al. (2002) showed that Australian women had twice the level of cadmium in their livers than did their male counterparts; they also noted a trend for higher cadmium content in the kidneys of the female subjects. Uetani et al. (2006) documented differences in cadmium accumulation in a range of tissues and organs between 72 men and women who lived in areas with no apparent cadmium pollution. In addition, several studies on human eyes have shown that the retinal pigment epithelium and choroids contained more cadmium than did the retina (Erie et al. 2005; Wills et al. 2008). These studies also found that women, men and women of older age, and smokers of both sexes had elevated levels of cadmium accumulation in eye tissues. Additional studies have demonstrated sex differences in ocular metal content in nondiseased eyes and those afflicted with AMD (Wills et al. 2008, 2009).

### Intestinal absorption of metals, body burden variability, and metal transporters

Highly efficient absorption, transport, and cellular uptake mechanisms have evolved in living organisms to ensure an optimal supply of essential metals. Such mechanisms are crucial for metals, because they cannot be synthesized or destroyed by the cells and must be mined from the external environment (Clemens 2006). As predicted from the U-shaped dose–response curve characteristics of essential metals, mechanisms designed to prevent deficiency or overdose toxicity have likely evolved for maintenance of homeostasis (Slikker et al. 2004). Cadmium has no known physiologic function, and no mechanism would have been expected to be evolved for its selective transport and homeostasis. In all likelihood, cadmium is acquired by transport mechanisms developed for essential metals. From physical and chemical properties, those metals are most likely to be zinc (Zn^2+^), iron (Fe^2+^), manganese (Mn^2+^), and calcium (Ca^2+^). In the literature, a considerable range defines the possible intestinal absorption rate for cadmium. For instance, it was estimated to be between 3 and 7% in humans and between 0.3 and 3.5% in rats. These values were used to assign an average 5% absorption rate in deriving a safe exposure level for cadmium (IPCS 1992; WHO 1989). However, higher cadmium absorption rates (20–40%) were shown in balanced studies (Horiguchi et al. 2004; Kikuchi et al. 2003). These studies also observed enhanced rates among young subjects and considered the possible biliary excretion and reuptake via enterohepatic circulation. Many investigators have shown the influence of body iron stores on absorption rate and body burden of cadmium. Satarug et al. (2004) found a 3- to 4-fold increase in cadmium body burden among Thai women who had low iron stores when compared with those of the same age and of normal iron stores. Kippler et al. (2007) found increased cadmium burden among Bangladeshi women associated with low iron stores only among those with adequate zinc status. An inverse correlation between serum iron and blood cadmium was observed among Canadian subjects: men had higher serum iron, blood lead, and serum selenium values than did women, and women had higher serum copper and blood manganese than did men (Clark et al. 2007; Copes et al. 2008). The higher blood manganese in women might be expected because low iron stores have been associated with enhanced manganese absorption (Finley 1999; Kippler et al. 2009). Some studies have shown no influence on body iron stores, but these were conducted in chronic high-exposure situations where metal transporters would likely be saturated with metal. Current data thus suggest metal transporters could be one of the determinants of cadmium body burden—a factor that may explain the variability in blood cadmium levels observed by Björkman et al. (2000) in a cohort of 61 monozygotic and 103 dizygotic twin pairs.

## Conclusions and Perspectives

Recent epidemiologic studies involving an exposure–effect assessment have linked low-level cadmium exposure of current populations with some adverse effects that are not restricted to kidney and bone, but include almost every organ and tissue where cadmium accumulates, including eye tissues. These data argue strongly for public health measures aimed at reducing exposure. In the past, the wide variation in cadmium body burden among people has been attributed to cigarette smoking and the high pulmonary absorption rates of cadmium in cigarette smoke. However, as revealed in the present review, the difference in body burden of cadmium between smokers and nonsmokers is less than 3-fold. We suggest that the signs of early renal injury and mild tubular impairment observed in chronic low-dose exposure situations viewed previously as benign could indeed be an early warning sign of subclinical or clinical morbidity and mortality. This assertion is substantiated by the dose response observed between cadmium body burden and all-cause mortality and cancer mortality in the Belgian and the U.S. cohorts. We also believe that cadmium is secreted in breast milk and that calcium and zinc supplements could be considered to lower the cadmium content in breast milk to minimize potential effects of early-life exposure to cadmium.

Many issues require further research. A precise risk estimate is needed to quantify the carcinogenic risk because the high prevalence of cadmium exposure means that even a small increase in risk could yield a large number of preventable cancer cases. To be valid, the threshold-based PTWI model, although appearing to be a reasonable method for deriving a safe exposure level, will require appropriate input from current scientific knowledge. Thus, revising the current safe intake level for cadmium is much needed. A strong consideration should be given to a safety factor issue, which is necessary to protect subpopulations with increased susceptibility, such as those with diabetes. Animal studies have shown that the symptoms of diabetic nephropathy and cadmium renal toxicity are enhanced when both the metal and the disease are present. The enhanced cadmium absorption noted for young age groups indicates that new intake guidelines may need to be established for pediatric populations. The application of the BMD method should be expanded and applied to other toxicity end points to identify the organs, other than the kidney, that should be considered as critical for deriving safe exposure levels. The potential genetically determined rates of cadmium absorption, uptake, accumulation, and toxicity remain largely unexplored and should be subjects of future research. With the looming cancer and chronic disease epidemics worldwide, we encourage research in the following areas: cadmium exposure assessment, identification of potential exposure sources, and the determination of cadmium body burden in future epidemiologic investigations. Such research would provide an estimate of total disease burden (cost) of population exposure. In addition, therapeutically effective chelating agents to enhance excretion of cadmium are lacking, and this factor makes prevention of cadmium accumulation pivotal. The persistence of cadmium in the environment requires a long-term approach to minimize human exposure through environmental management and maintenance of lower cadmium levels wherever possible.

## Figures and Tables

**Table 1:** Exposure levels associated with kidney and bone effects.

Study population, age, reference	Exposure/outcomes
Sweden, *n* = 820, 53–64 years of age, Åkesson et al. 2005, 2006	Blood and urinary cadmium at 0.38 μg/L and 0.67 μg/g creatinine were associated with tubular impairment. Urinary cadmium at 0.8 μg/g creatinine was associated with glomerular impairment. Increased body burden of cadmium was associated with lowered bone mineral density, decreased serum parathyroid hormone and bone metabolism.

Thailand, *n* = 200, 16–60 years of age, Satarug et al. 2005	A 3-fold increase in body burden associated with 11%, 32%, and 61% increases the probability of having high blood pressure, renal injury, and tubular impairment.

Thailand, *n* = 224, 30–87 years of age, Teeyakasem et al. 2007	OR for tubular impairment was 10.6, comparing urinary cadmium 1–5 versus > 5 μg/g creatinine.

United States, *n* = 4,258, ≥ 50 years of age, Gallagher et al. 2008	A 1.43-fold increase in osteoporosis risk, comparing urinary cadmium 1 versus < 0.5 μg/g creatinine

Belgium, *n* = 294, mean age 49.2 years of age, Schutte et al. 2008b	A 2-fold increase in body burden associated with increased bone resorption, urinary calcium loss, decreased proximal forearm bone density, and low serum parathyroid hormone.

China, *n* = 148, 3-year observation, Wu et al. 2008	Progressive tubular and glomerular impairment was observed among those with urinary cadmium > 10 μg/g creatinine.

United Kingdom, *n* = 160, 18–86 years of age, Thomas et al. 2009	Risk for early renal effects^a^ was increased by 2.6-fold and 3.6-fold, comparing urinary cadmium 0.3 versus < 0.5 versus ≥ 0.5 μg/g creatinine.

United States, *n* = 14,778, > 20 years of age, Navas-Acien et al. 2009	Risk for albuminuria was 2.34 and risk for lowered glomerular filtration rate was 1.98, comparing those in the highest versus lowest quartiles of blood cadmium and lead.

OR, odds ratio.

a Early renal injury was defined as urinary NAG > 2 IU/g creatinine.

**Table 2:** Exposure levels associated with diabetes and hypertension.

Study population, age, reference	Exposure/outcomes
United States, *n* = 8,722, ≥ 40 years of age; Schwartz et al. 2003	OR for abnormal fasting glucose was 1.48, 2.05, comparing urinary cadmium < 1 versus 1.00–1.99 versus ≥ 2 μg/g creatinine, respectively. OR for diabetes was 1.24, 1.45, comparing urinary cadmium < 1 versus 1.00–1.99 versus ≥ 2 μg/g creatinine, respectively.

China, *n* = 229, 44–87 years of age with type 2 diabetes, mean diabetic duration 8.6 years; Chen et al. 2006	OR for tubular impairment was 3.34, comparing urinary cadmium < 1 versus ≥ 1 μg/g creatinine; it was increased to 5.56, comparing those with low versus high levels of circulating metallothionein antibody.

Pakistan, *n* = 238 men, 31–60 years of age with type 2 diabetes, diabetic duration 16 years, 196 controls; Afridi et al. 2008	Subjects with diabetes had higher levels of cadmium in hair, blood, and urine than did controls. Mean blood (urinary) cadmium was 4.2 (3.2) μg/L among nonsmoker controls and 5.7 (4.6) μg/L among nonsmoker cases.

Torres Strait, Australia, *n* = 182; Haswell-Elkins et al. 2008	A dose response between urinary cadmium and glomerular impairment was observed among subjects with type 2 diabetes after adjusting for confounders.

Korea, *n* = 1,902; Eum et al. 2008	OR for hypertension was 1.51, comparing blood cadmium in the lowest versus the highest tertile.

United States, *n* = 10,991 ≥ 20 years of age; Tellez-Plaza et al. 2008	Mean difference in systolic blood pressure between blood cadmium in the 90th versus 10th percentile was 1.36 mmHg (95% CI, –0.28 to 3.00), whereas the mean difference in diastolic blood pressure was 1.68 mmHg (95% CI, 0.57 to 2.78).

OR, odds ratio.

**Table 3:** Exposure levels associated with effects on newly identified targets.

Targets/study population, reference	Exposure/outcomes
Blood vessels: United States, *n* = 2,125, Navas-Acien et al. 2004; *n* = 790, Navas-Acien et al. 2005	OR for PAD of 1.07, 1.30, and 2.82, when comparing blood cadmium quartiles 2, 3, and 4 versus the lowest (*p* for trend = 0.01). OR for PAD of 3.05, when comparing urinary cadmium of the 75th versus the 25th percentile.

Blood vessels: Belgium, *n* = 557; Schutte et al. 2008a	Increased body burden associated with lower aortic pulse wave velocity, lower pulse pressure, and higher femoral distensibility.

Heart: United States, *n* = 4,912; Everett and Frithsen 2008	OR for female myocardial infarction was 1.8, comparing urinary cadmium ≥ 0.88 versus < 0.43 μg/g creatinine.

Lung: United States, *n* = 96; Lampe et al. 2008	Increased body burden was associated with reduced lung function among smokers.

Periodontal tissues: United States, *n* = 11,412; Arora et al. 2009	A 3-fold increase in urinary cadmium associated with 54% higher prevalence odds for periodontal disease.

Eye: United States, *n* = 53 cases, 53 controls; Erie et al. 2007	Higher urinary cadmium associated with AMD in smokers.

Mammary gland: Austria, *n* = 124; Gundacker et al. 2007	Intake of supplement was associated with lowered breast milk cadmium only in nonsmokers.

Mammary gland: Bangladesh, *n* = 123; Kippler et al. 2008	Manganese, iron, and calcium in breast milk correlated with cadmium content.

OR, odds ratio.

**Table 4:** Exposure levels associated with mortality and cancer mortality.

Study population, reference	Exposure/outcomes
Kakehashi (Japan) cohort, *n* = 3,178, 15-year observation; Nakagawa et al. 2006; Nishijo et al. 2006	Hazard ratio for cancer mortality was 2.5 among women with permanent tubular impairment.^a^ Hazard ratio for all-cause mortality was 2.09 among women with urinary cadmium ≥ 3 μg/g creatinine.

Nagasaki (Japan) cohort I, *n* = 275, 23-year observation; Arisawa et al. 2007a	OR for cancer mortality was 2.58 among those with tubular impairment.^a^ OR for all-cause mortality was 1.41 among those with permanent tubular impairment.^a^
Nagasaki cohort II, *n* = 329, 13-year observation; Arisawa et al. 2007b	No effects of body burden of cadmium on mortality were observed.

Belgian cohort, *n* = 956, 20.3-year median observation; Nawrot et al. 2008	Mortality increased by 20% and 44% in low- and high-exposure areas, respectively, among those with a 2-fold increase in body burden. Mortality increased by 25% and 33% in low- and high-exposure areas, respectively, among those with a 2-fold increase in blood cadmium.

U.S. cohort, *n* = 13,958, Menke et al. 2009	Male hazard ratio was 1.7 for all-cause mortality and 4.3 for cancer mortality, comparing urinary cadmium < 0.21 versus > 0.48 μg/g creatinine.

OR, odds ratio.

a Irreversible tubular impairment was defined as urinary β2-MG ≥ 1,000 μg/g creatinine.

**Table 5:** Exposure levels associated with cancer.

Cancer/study population, reference	Exposure/risk estimate
Lung, Belgium, *n* = 994, 15-year observation; Nawrot et al. 2006	Hazard ratios of 1.7, 2.6, and 1.6 were attributed to a 2-fold increase in body burden, living in high-exposure area, and a 2-fold increase in soil cadmium, respectively.

Pancreas, Egypt, *n* = 31 cases, 52 controls; Kriegel et al. 2006	ORs of 1.12 and 3.25 were attributed to elevated serum cadmium and farming occupation, respectively.

Breast, United States, *n* = 246 cases, 254 controls; McElroy et al. 2006	OR of 2.3 when comparing urinary cadmium < 0.26 versus ≥ 0.58 μg/g creatinine

Endometrium, Sweden, *n* = 30,210, 16-year observation; Åkesson et al. 2008	OR of 2.9 was attributed to cadmium intake > 15 μg/day.

Prostate, China; *n* = 297, Zeng et al. 2004	Dose response between body burden and abnormal serum PSA levels

Prostate, Italy, *n* = 45 cases, 58 controls; Vinceti et al. 2007	OR of 4.7 when comparing nail cadmium content in the lowest versus the highest quartile

Prostate, United States, *n* = 422; Wijngaarden et al. 2008	An increase of urinary cadmium to 1 μg/g creatinine associated with a 35% increase in serum PSA

Urinary bladder, Belgium, *n* = 172 cases, 395 controls; Kellen et al. 2007	OR of 5.7 when comparing blood cadmium in the lowest versus the highest tertile

Abbreviations: OR, odds ratio; PSA, prostate-specific antigen.

**Table 6:** Cadmium accumulation in the body of environmentally exposed subjects.

Study population, reference	Cadmium content (μg/g wet tissue weight)	
	Men	Women
Australia, Satarug et al. 2002^a^		
Lung	0.11 ± 0.19	0.17 ± 0.35
Liver	0.78 ± 0.71	1.36 ± 0.96^b^
Kidney cortex	14.6 ± 12.4	18.1 ± 18.0
Japan, Uetani et al. 2006^c^		
Liver	7.9 (2.1)	13.1 (2.1)
Kidney cortex	72.1 (1.7)	83.9 (2.2)
Kidney medulla	18.3 (2.2)	24.5 (2.1)
Pancreas	7.4 (2.0)	10.5 (2.1)
Thyroid	10.6 (2.2)	11.9 (2.0)
Heart	0.3 (1.5)	0.4 (2.0)
Muscle	1.2 (2.1)	2.2 (2.4)
Aorta	1.0 (2.1)	1.1 (1.9)
Bone	0.4 (1.6)	0.6 (1.8)

a An Australian study comprising 43 men and 18 women, 2–89 years of age (mean age, 38.5 years). Values are arithmetic mean ± SD.

b Higher in women than in men.

c A Japanese study comprising 36 men and 36 women, 60–91 years of age (mean age, 74 years). Values are geometric mean (SD).
